# Nitrite Reductase 1 Is a Target of Nitric Oxide-Mediated Post-Translational Modifications and Controls Nitrogen Flux and Growth in Arabidopsis

**DOI:** 10.3390/ijms21197270

**Published:** 2020-10-01

**Authors:** Álvaro Costa-Broseta, MariCruz Castillo, José León

**Affiliations:** Instituto de Biología Molecular y Celular de Plantas, Consejo Superior de Investigaciones Científicas—Universidad Politécnica de Valencia, 46022 Valencia, Spain; tyuhan@hotmail.com (Á.C.-B.); macaslo3@ibmcp.upv.es (M.C.)

**Keywords:** CRISPR, cysteine S-nitrosylation, nitrate assimilation, nitric oxide, nitrite reductase, tyrosine nitration, plant growth, ubiquitylation

## Abstract

Plant growth is the result of the coordinated photosynthesis-mediated assimilation of oxidized forms of C, N and S. Nitrate is the predominant N source in soils and its reductive assimilation requires the successive activities of soluble cytosolic NADH-nitrate reductases (NR) and plastid stroma ferredoxin-nitrite reductases (NiR) allowing the conversion of nitrate to nitrite and then to ammonium. However, nitrite, instead of being reduced to ammonium in plastids, can be reduced to nitric oxide (NO) in mitochondria, through a process that is relevant under hypoxic conditions, or in the cytoplasm, through a side-reaction catalyzed by NRs. We use a loss-of-function approach, based on CRISPR/Cas9-mediated genetic edition, and gain-of-function, using transgenic overexpressing HA-tagged Arabidopsis NiR1 to characterize the role of this enzyme in controlling plant growth, and to propose that the NO-related post-translational modifications, by S-nitrosylation of key C residues, might inactivate NiR1 under stress conditions. NiR1 seems to be a key target in regulating nitrogen assimilation and NO homeostasis, being relevant to the control of both plant growth and performance under stress conditions. Because most higher plants including crops have a single NiR, the modulation of its function might represent a relevant target for agrobiotechnological purposes.

## 1. Introduction

Plant growth is the result of the coordinated photosynthesis-mediated assimilation of oxidized forms of C, N and S. These reductive processes end primarily with the biosynthesis of amino acids and nucleotides, which allow the further synthesis of proteins and nucleic acids that is essential for plant growth and development. Nitrate is the predominant N source in soils and its reductive assimilation requires the successive activities of soluble cytosolic Nicotinamide adenine dinucleotide (NADH)-nitrate reductases (NR) [1] and plastid stroma ferredoxin-nitrite reductases (NiR) [2] allowing the conversion of nitrate to nitrite and then to ammonium, thus also avoiding the elevated toxicity of nitrite [3]. The model plant *Arabidopsis thaliana* has two NADH-dependent nitrate reductases, NIA1 and NIA2, but a single nitrite reductase called NiR1 [4]. Ammonium is further incorporated to glutamate and glutamine through the plastidic glutamine synthetase/glutamate synthase (GS/GOGAT) cycle [5]. This reductive assimilatory pathway thus involves different subcellular locations as it begins in the cytoplasm but ends in chloroplasts. However, nitrite, instead of being reduced to ammonium in plastids, can be reduced to nitric oxide (NO) in mitochondria, through a process that is relevant under hypoxic conditions [6], or in the cytoplasm, through a side-reaction catalyzed by NRs [7,8]. The balance between redox reactions catalyzed by NR and NiR then seems crucial to determine the amount of NO that plants can produce and accumulate.

NO has been extensively characterized as a regulator of plant growth and development as well as multiple responses to environmental stress [9], so knowledge on the way it is produced and acts is crucial to control plant performance. NO’s mode of action as a signaling molecule largely relies on its nature as a free radical and its capacity to react with macromolecules, particularly with specific amino acids of proteins, thus leading to nitrated or S-nitrosylated proteins [10,11].

Here we perform a functional characterization of the *Arabidopsis thaliana* nitrite reductase 1 (NiR1) based on two complementary approaches. First, the Clustered Regularly Interspaced Short Palindromic Repeats (CRISPR)/Cas9-mediated generation of a *nir1* knockout mutant points to a key role in regulating the flux of nitrogen to ammonium and NO, and thus controlling growth. Second, immunopurification of protein extracts from HA-tagged *NiR1* overexpressing plants followed by MS/MS allowed the identification of NO-related post-translational modifications of NiR1 that may be responsible for the inactivation of the enzyme under NO-related stress conditions.

## 2. Results

### 2.1. Generation of a NiR1 Mutant by CRISPR/Cas9-Mediated Genetic Edition

**Figure 1:**
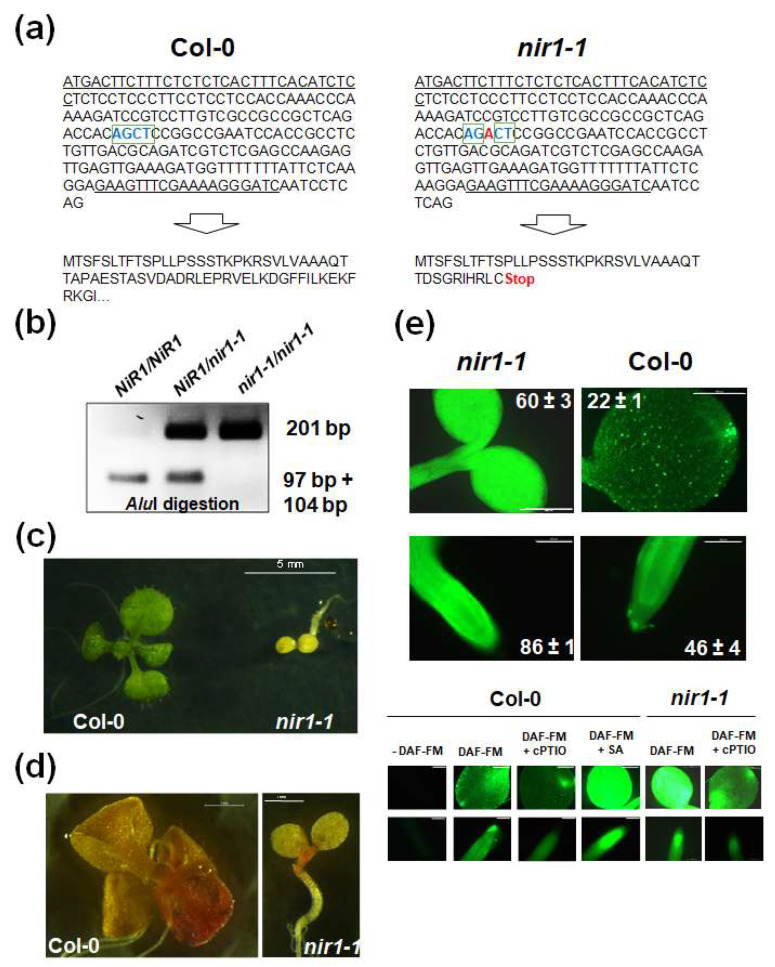
Generation of *nir1-1* mutant plants by CRISPR/Cas9 gene editing. (**a**) 5′sequence of the *NiR1* locus showing the A (red) insertion generating a Restriction Fragment Length Polymorphism with the disruption of an *Alu*I restriction site and, as shown in the translated amino acid sequences, the generation of a premature stop codon in the mutant plants. (**b**) PCR-assisted genotyping of progeny using the primers corresponding to the underlined sequences in panel a. The ethidium bromide stained 2% agarose gel is shown (**c**) Extreme stunted and chlorotic phenotype shown by homozygous *nir1-1* plants. (**d**) Nitrite staining after treatment with sulfanilamide and N-(1-naphtyl)-ethylenediamine. (**e**) Endogenous levels of NO in wild type Col-0 and *nir1-1* mutant plants. Images are representative of cotyledons (upper panels) and roots (lower panels) of each genotype and condition tested after treatment with 4-Amino-5-Methylamino-2′,7′-Difluorofluorescein Diacetate (DAF-FM diacetate). Images were captured with a Leica DM 5000B fluorescence microscope equipped with a chlorophyll auto fluorescence filter and digital camera. Scale bars correspond to 100 and 500 μm in roots and cotyledons, respectively. Values are the mean ± standard error of four independent replicates.

*Arabidopsis thaliana*, as a model plant, has contributed decisively to knowledge of the function of plant enzymes through direct and reverse genetic approaches. However, the fact that Arabidopsis has a single NiR called NiR1, and that this enzyme catalyzes a key essential step in nitrogen assimilation, has so far likely precluded the identification and characterization of mutants in this gene. We searched public databases for mutants in the *NiR1* locus (AT2G15620) and found no positive results. We thus decided to follow an alternative way to obtain plants with reduced *NiR1* expression, through CRISPR/Cas9-mediated genetic edition using as a guide the 19 bp GCCGCTCAGACCACAGCTC target sequence located close to the 5′-end of the NiR1 locus. The target site was selected according to the GC content and location in the gene sequence using the website eCRISP (http://www.e-crisp.org/E-CRISP/ German Cancer Research Center DKFZ, Germany) checking for off-target = 0 to avoid secondary targets. A protocol using Site-directed, Ligase-Independent Mutagenesis (SLIM) and further subcloning in the pHEE2E-TRI destination vector, which contains CRISPR/Cas9 under the control of an egg cell-specific promoter, was performed. The sequence verified construct was used to transform *Agrobacterium tumefaciens* C58 and these were further used to genetically transform *Arabidopsis thaliana* Col-0 plants. A total of 172 hygromycin resistant T1 seedlings transformed with *pHEE2E-gNIR1*, carrying both the Cas9 gene under the control of the Egg Cell Promoter and the RNA scaffold with the RNA guide for *NiR1* under the control of the *U6* promoter, were genotyped by sequencing the region around the RNA guide target in the *NiR1* locus. Among 172 sequenced T1 individuals, 51% were wild type plants and 49% were heterozygous mutant plants. No T1 plant carried a homozygous mutation. Moreover, 46% of the sequenced T1 plants carried a 1 bp insertion and only five individuals (3%) carried a 10 bp insertion. Among the 1 bp insertions, 67 out of 79 plants displayed an A insertion (Figure 1a). This selected mutation, which we called *nir1-1*, represents to our knowledge the first reported nitrite reductase Arabidopsis mutant. It consisted of a single A base insertion in position 98 causing a frame shift, leading to a premature stop codon, and a Restriction Fragment Length Polymorphism (RFLP), because of disruption of the *Alu*I restriction site present in wild type and therefore absent in mutant plants (Figure 1b).

Homozygous *nir1-1* mutant plants, selected in the second generation (Figure 1b), displayed a strong stunted growth phenotype and chlorosis even when grown with ammonium nitrate as the nitrogen source (Figure 1c). This phenotype was not accompanied by a strong accumulation of nitrite as would be expected for NiR1 loss of function (Figure 1d), thus suggesting that nitrite should be metabolized to a metabolite different than ammonium. We analyzed the NO endogenous content of *nir1-1* plants by using DAF-FM diacetate fluorophore and found a strong fluorescence accumulation in cotyledons and roots over levels detected in wild type plants (Figure 1e). Fluorescence was confirmed to be due to NO accumulation, as plants incubated with DAF-FM and the NO-inducer salicylic acid (SA )showed enhanced fluorescence levels, whereas treatment with the NO scavenger 2-(4-Carboxyphenyl)-4,4,5,5-tetramethylimidazoline-1-oxyl-3-oxide (cPTIO) displayed lower levels of fluorescence (Figure 1e).

### 2.2. NiR1 Is Post-Translationally Modified in Planta by NO-Triggered Nitration and S-Nitrosylation

**Figure 2:**
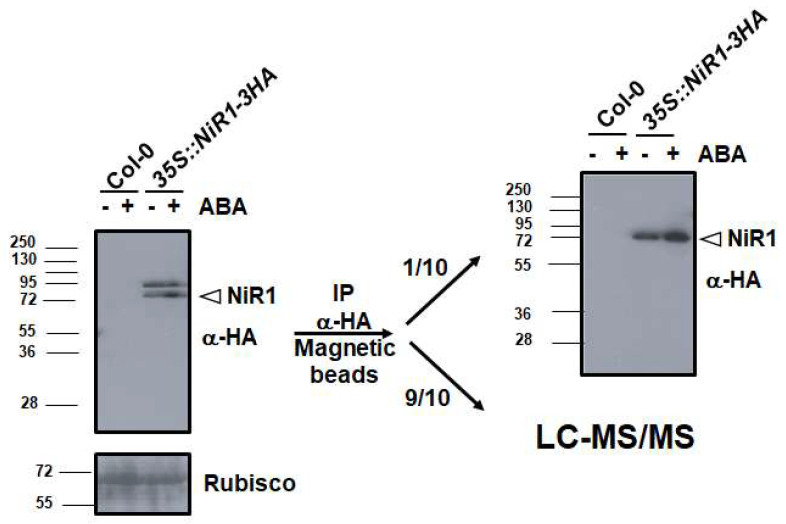
*35S::NiR1-3HA* plants as a tool for NiR1 immunopurification and subsequent proteomic analyses. Extracts from wild-type Col-0 and transgenic plants either untreated (−) or treated with 50 μM ABA for three hours (+) were analyzed by Western blot with anti-HA antibody (left panel). Ponceau S staining of the Rubisco protein is included below as loading control. Protein extracts were further immunopurified with anti-HA-coated magnetic beads. A fraction of the immunopurified NiR1 was checked by Western blot with anti-HA antibody (right panel) and the rest of the protein samples (untreated and ABA-treated) were further analyzed by LC-MS/MS.

**Figure 3:**
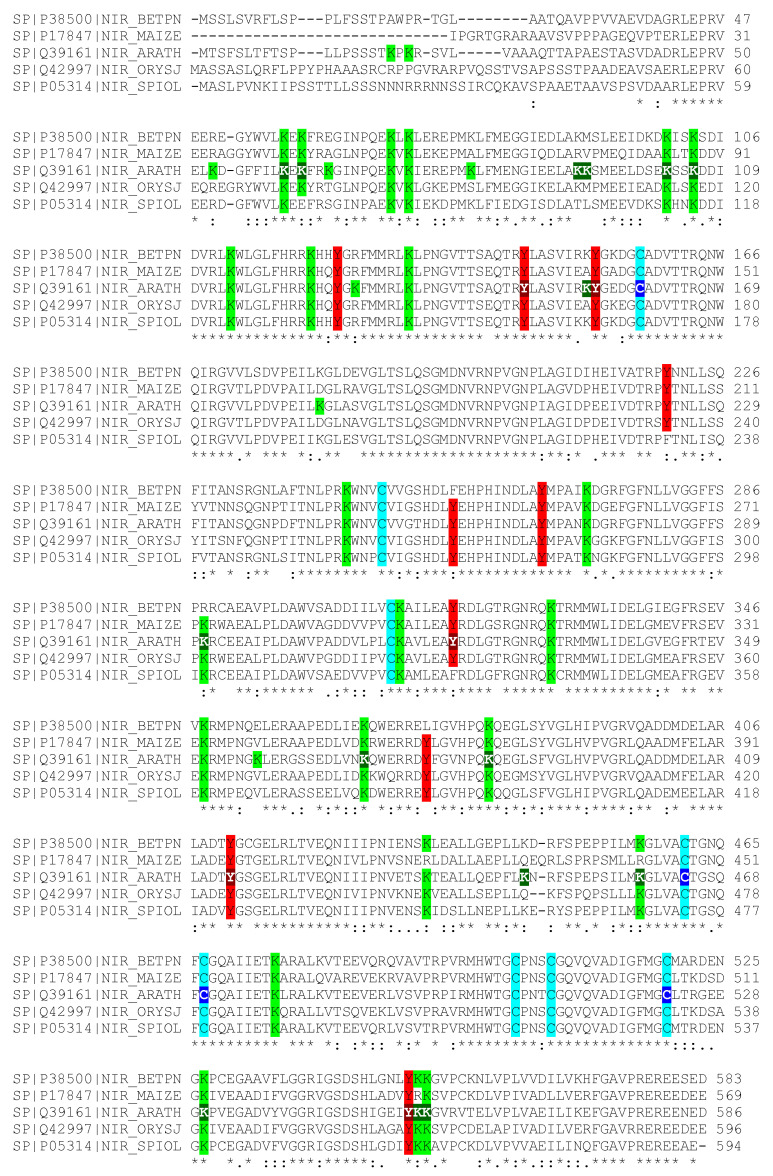
Post-translational modifications identified in planta for Arabidopsis NiR1. A, Multiple aligned amino acid sequences of different plant NiRs (silver birch BETPN; corn MAIZE; Arabidopsis thaliana ARATH; rice ORYSJ; and, spinach SPIOL) showing conserved residues highlighted in green, red and blue for K, Y and C, respectively. The Arabidopsis NiR1 tyrosine (Y), cysteine (C), and lysine (K) residues that were identified as nitrated, S-nitrosylated and ubiquitylated are shown in white characters on dark red, blue and green backgrounds, respectively. *,: and. represent fully conserved residues, and residues with similarity score >0.5 and <0.5, respectively

**Figure 4:**
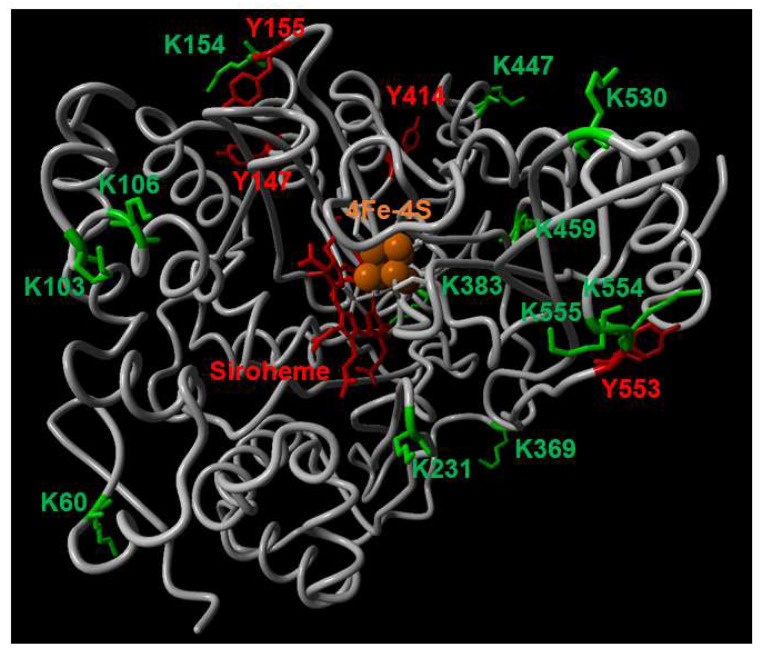
Location of identified PTMs in the 3D structure model of Spinacia oleracea NiR. Nitrated Y residues (red) and ubiquitylated K residues (green) location in the 3D structure of spinach NiR (2akj.pdb Protein Database Bank code) displayed in gray. Molecules of the 4Fe-4S cluster and siroheme are colored in orange and red, respectively.

**Figure 5:**
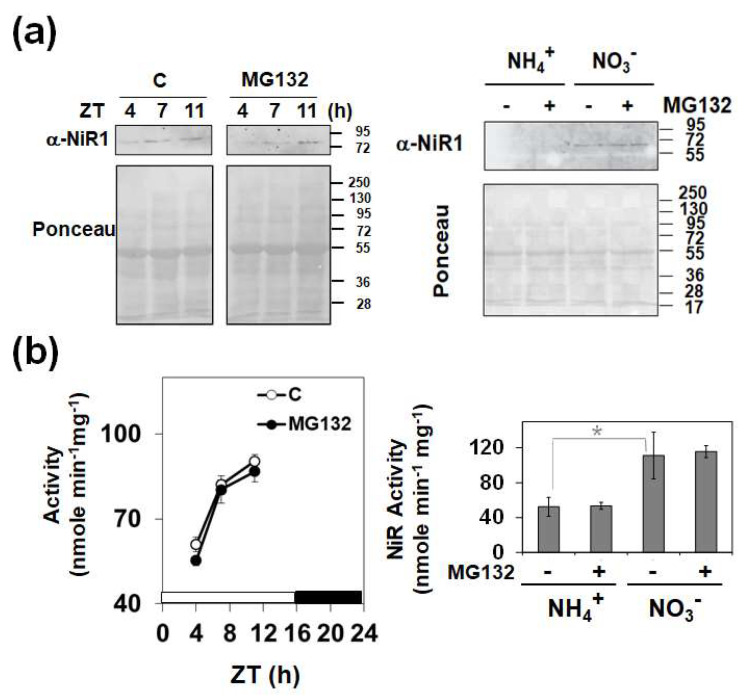
Effect of proteasome-mediated degradation on light- and nitrate-induced NiR1 protein and enzyme activity. (**a**) NiR1 protein levels and (**b**) NiR1 activity in long day-photoperiodic conditions for 11 days and collected either at the indicated Zeitgeber time (ZT) during the light phase (left panels) or by growing for 9 days in ammonium-supplied media and then transferred to media containing either 10 mM nitrate (NO_3_^−^) or 5 mM ammonium (NH_4_^+^), as indicated, for two additional days (right panels). By 16 h before collecting samples at either different zeitgeber time (ZT) (left panels) or before and after nitrate supply (right panels), plants were either untreated (−) or treated with 100 μM MG132 proteasome inhibitor (+) as indicated. Panels in (**a**) show representative Western blots obtained by using polyclonal anti-NiR1 antibodies. Equal protein loading was assessed by Ponceau S staining of the blot. The position of molecular mass markers is shown at the right side of the blots. Values of enzyme activity are the mean ± standard error of three independent biological replicates. * Represents statistical significance with *p* < 0.05 in the paired Student’s *t*-test.

**Figure 6:**
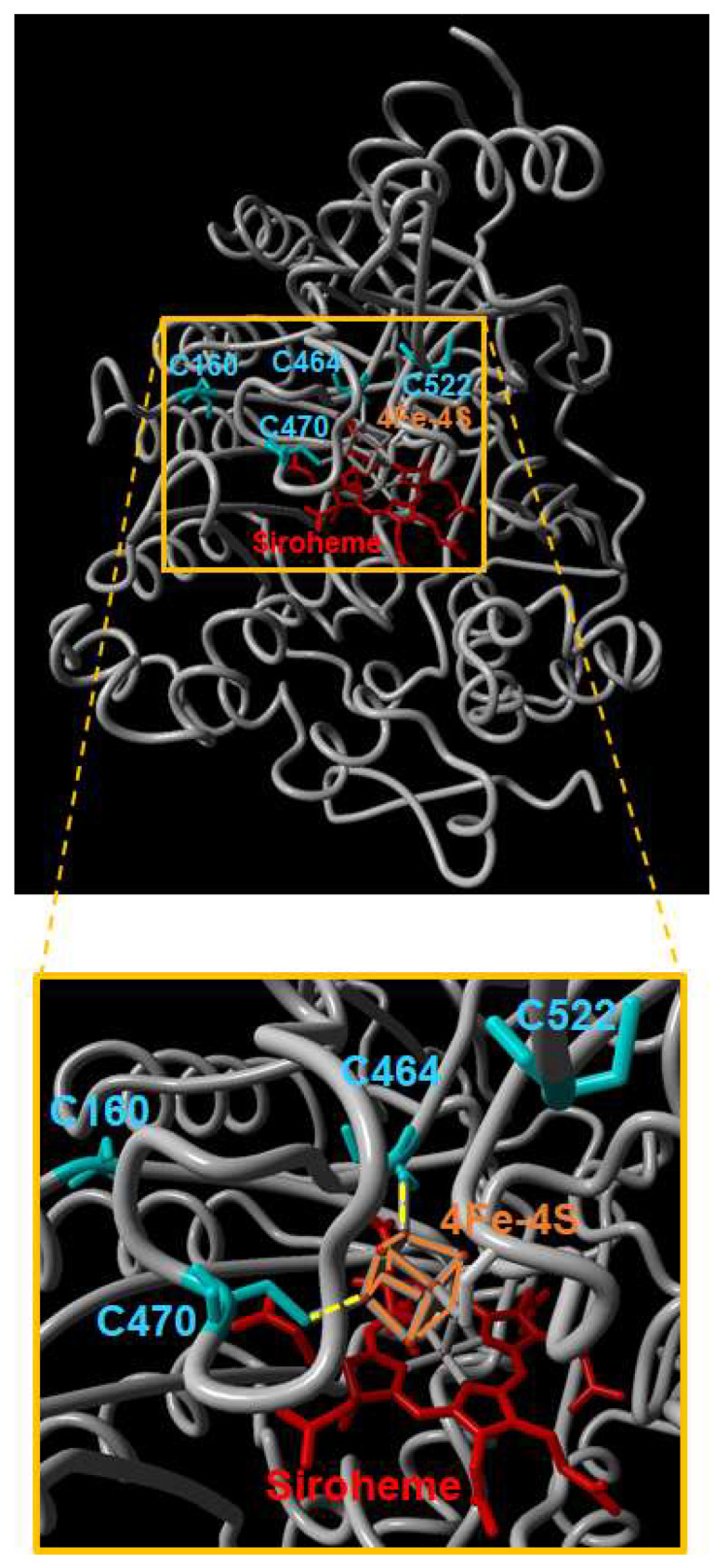
Location of S-nitrosylated cysteines in the 3D model of NiR1. The NiR1 3D structure was modelled in the basis of the 3D structure obtained from spinach ferredoxin-nitrite reductase crystals (PDB code 2akj) using the Yasara application (www.yasara.org). The position of the siroheme (red) and 4Fe-4S cluster (orange) cofactors together with each modified amino acid are shown. Dashed yellow lines in the bottom panel represent the bonds between C473 and C479 and the 4Fe-4S cluster.

Because the NiR1 loss-of-function strongly induced NO accumulation and severely impaired growth, stress-related physiological conditions involving reduced nitrite reductase activity might be likely to be relevant to the control of plant growth and development. To perform a molecular analysis on whether NiR1 protein activity may be regulated by NO-related factors, we generated transgenic lines over-expressing hemagglutinin (HA)-tagged versions of NiR1 (Figure 2). Protein extracts from *35S::NiR-3HA* plants, under abscisic acid (ABA)-induced accumulation of NO, were used for further immunopurification with anti-HA-coated magnetic beads. Proteomic methodologies based on LC-MS/MS techniques made it possible to identify that NiR1 undergo multiple post-translational modifications (PTMs) including ubiquitylation of K residues, S-nitrosylation of C residues and nitration or amination of Y residues (Supplementary Table S1). Four out of five nitrated Y residues identified (Y147, 155, 414 and 553) were fully conserved in NiRs from other plants, and the most robustly identified nitrated Y319, with eight peptides identified as nitrated in the proteomic analyses (Table S1), was conserved in all but spinach NiR (Figure 3). Taking advantage of the homology between the spinach and Arabidopsis NiRs and the reported 3D structure of spinach NiR (Protein Database Code 2akj), we have located the equivalent spinach residues to that identified as modified in Arabidopsis NiR1 (Figure 4). All these nitrated Y residues were in the outermost part of the structure, far from the 4Fe-4S cluster and siroheme binding pocket (Figure 4). Regarding ubiquitylated K residues, close to 50% of the K residues were found to carry the GG tag after trypsin proteolysis of the ubiquitin chains in our proteomic analyses (Figure 3). These data are consistent with the in silico prediction of ubiquitylation for NiR1 protein showing that 20 out of 37 K residues were potentially ubiquitylated (Table S2). Only four out of 15 identified ubiquitylated K were not fully conserved amino acids in plant NiRs (Figure 3). As shown above for the nitrated Y, ubiquitylated K were also located far from the cofactor binding sites of the enzyme, at the most exposed external part of the protein (Figure 4). Extensive ubiquitylation suggest that NiR1 protein might be regulated through polyubiquitylation-triggered proteasomal degradation. We checked whether NiR1 protein stability might be regulated by proteasomal degradation. NiR1 protein and enzyme activity increased during the day phase along photoperiod or by supplying nitrate as the N source, and the levels of activity and protein were not significantly different in plants pre-treated with the proteasome inhibitor MG132 in any of those NiR1 inducing conditions (Figure 5). Therefore, the ubiquitylation pattern detected in NiR1 seems not to be related with proteasome-mediated regulation by proteolysis. We also found that four out of eight fully conserved C amino acids of NiRs were identified as S-nitrosylated (Table S1 and Figure 3). As shown in Figure 6 using the spinach NiR as structural model, S-nitrosylated C160 and C522 were far from the cofactor binding pocket and oriented towards the protein surface. In contrast, S-nitrosylated C464 and C470 were very close and oriented to the cofactor binding pocket (Figure 6). Actually, C470 would be involved in binding 4Fe-4S cluster. The S-nitrosylation of the cysteines involved in binding the 4Fe-4S cluster in the catalytic center of NiR1 is very likely impairing the cofactor binding, and thus affecting the proper electron transference and the activity of the modified enzyme.

## 3. Discussion

The characterization of *nir1-1* plants has made it possible to identify that the inability of reducing nitrite to ammonium caused an increase in NO content instead of nitrite accumulation. These results allow ruling out a direct involvement of NiR1 in NO biosynthesis but point to this enzyme as a relevant target for controlling NO homeostasis. Further phenotypic characterization of *nir1-1* plants pointed to an essential requirement of ammonium production for growth as well as a toxic effect caused by the overproduction of NO, as reflected by the chlorotic stunted phenotype of plants. Moreover, *nir1-1* plants displayed very slow growth in the early post-germination stage and strongly reduced seedling establishment when cultivated with nitrate and ammonium as N sources, presumably because the use of nitrate leads mostly to a large accumulation of NO with detrimental effects on growth. In the eukaryotic algae *Chlamydomonas reinhardtii*, ammonium and NO strongly repress nitrate assimilation by repressing the expression and inhibiting the activity of key enzymes and transporters of the pathway [12,13]. However, in Arabidopsis, ammonium seems to activate the expression of one of the two nitrate reductase encoding genes by repressing its methylation [14]. On the other hand, elevated levels of endogenous NO or exogenous application have been correlated with reduced hypocotyl and root growth in Arabidopsis [15,16]. This situation characterized in Arabidopsis may be relevant for some plants including important crops, as their genomes also contain a single gene coding for NiR1 orthologs (Table S3 and Figure S1). However, it is worth mentioning that some important crops such as rice, maize, potato, tomato, soybean, poplar and Brassica have two orthologues (Table S3 and Figure S1), thus introducing potential functional redundancy that makes NiRs less decisive in controlling plant performance. Our results indicate that in plants with a single NiR, nitrite reduction to ammonium is likely a key step not only for proper nitrogen assimilation but also for NO production, both factors being relevant for determining plant growth rates. NO has been also extensively characterized as a regulator of plant responses to abiotic and biotic stress factors [8]. Therefore, the altered function of NiR1 in Arabidopsis and in plants with single NiR may have also an impact on plant defense to stress.

The relevance of regulating nitrite reductase activity for controlling plant growth and defense responses point to physiological conditions potentially affecting NiR1 function. In this work, we analyzed mechanisms based on post-translational modifications of NiR1 potentially altering the stability and/or function of the enzyme. An IP-MS/MS proteomic analysis with protein extracts from transgenic plants overexpressing HA-tagged NiR1 allowed the discovery of an extensive pattern of lysine ubiquitylation all over the protein, as well as several nitrated tyrosines and S-nitrosylated cysteines mostly conserved in plant NiRs. K can be monoubiquitylated or polyubiquitylated and both modifications may alter both the subcellular localization and the stability of the modified proteins [17]. Polyubiquitylation usually marks target proteins for proteasome-mediated proteolytic degradation [18]. Although we found ubiquitylated NiR1, it is unlikely that ubiquitylation-mediated proteolysis of NiR1 represents a relevant factor in controlling protein stability, as we did not find significant effects in either protein stability or nitrite reductase activity in plants treated with a proteasome inhibitor (Figure 5). It is worth mentioning that polyubiquitylation occurs in the cytoplasm and NiR1 is a chloroplastic enzyme [2], so the process of ubiquitylation must occur before transit into plastids [19]. It would be also feasible that ubiquitylation of NiR1 does not alter its stability but its subcellular localization, hampering the transit to plastids and thus disconnecting it from the GS/GOGAT cycle enzymes catalyzing the incorporation of ammonium to carbon compounds.

The effect of NO-related PTMs on NiR1 function is much more likely. The cysteines involved in binding the 4Fe-4S cluster were found to be S-nitrosylated (Figure 6). Modification of the thiol group of cysteines would directly hamper the efficient binding of the cluster, which is essential for the proper sequential electron transfer from the reduced ferredoxin to 4Fe-4S cluster and siroheme of NiR1 [20,21]. Therefore, NO could induce NiR1 inactivation through S-nitrosylation of these critical cysteine residues. This mechanism would allow NO to potentiate its own biosynthesis, by preventing nitrite reduction to ammonium, a process that we have confirmed occurs in Arabidopsis *nir1-1* mutant plants (Figure 1), and it has been also reported in NiR-antisense tobacco plants [22]. This mechanism would allow a positive regulatory loop exerted by NO on plants to slow growth and potentiate defenses in transient responses to stress conditions.

## 4. Materials and Methods

### 4.1. Plant Material

Arabidopsis wild type Col-0 seeds were surface sterilized with chlorine gas before sowing in MS media plates containing 1% sucrose. Plants over-expressing HA-tagged NiR1 were generated by subcloning the full-length cDNA in pAlligator 2 vector, and further transformation of *Agrobacterium tumefaciens* with the corresponding construct. Plants were then genetically transformed by dipping floral organs in a suspension of transformed Agrobacterium [23].

### 4.2. Generation of NiR1-1 Mutant By CRISPR/Cas9 Technology

The NiR1 specific guide was subcloned in the pDONR207-Cas9 vector by using Site-directed, Ligase-Independent Mutagenesis (SLIM) [24] with two separate inverse PCRs using tailed primers (Table S4) and subsequent hybridization (Figure S2a). Gene targeting was performed with egg cell-specific promoter-controlled CRISPR/Cas9 technology by using the egg cell-specific promoter pHEE2E-TRI vector [25]. Mutation in *nir1-1* consisted of a single A base insertion in position 98 causing a frame shift leading to a premature stop codon (Figure S2b). Genotyping was performed by PCR amplification with specific primers (Table S4) and subsequent digestion with the restriction enzyme *Alu*I, which cuts the wild type but not the mutant amplicon.

### 4.3. Western Blot Analyses

The levels of NiR1 protein were analyzed in total protein extracts by SDS-PAGE, semi-dry blotting onto nitrocellulose membranes and further probing with polyclonal anti-NiR1 (1:1000 dilution) [26] or monoclonal anti-HA-Horseradish peroxidase (HRP) (1:1000 dilution) for *35S::NiR1-3HA* transgenic lines. Loading control was assessed by staining nitrocellulose membranes after blotting with Ponceau S.

### 4.4. Nitrite Reductase Activity Assay

Nitrite reductase activities were assayed as previously reported [27] with slight modifications. Assays included 50 μg of protein extracts in 250 μL total volume by using dithionite-reduced methyl viologen as an electron donor. Nitrite was colorimetrically determined by reaction with a 1:1 solution of sulfanilamide:N-(1-naphthyl)-ethylene-diamine dihydrochloride (NNEDA). Modifications of NiR activity assays were done to adapt volumes to microplate reading devices.

### 4.5. Proteomic Analyses of HA-Tagged Overexpressing Plants

Total crude protein extracts were prepared by grinding liquid nitrogen frozen samples and further extraction in 50 mM Tris-HCl buffer, pH 8.0, containing 150 mM NaCl, 5% glycerol, 5 mM EDTA and 0.05% (*v*/*v*) Triton X-100. Immunopurification with anti-HA-magnetic beads (Miltenyi Biotec, Gladbach, Germany) and elution under non-denaturing non-reducing conditions as well as LC-MS/MS-based proteomic analyses were performed as previously reported [28]. Proteins extracts were obtained from plants expressing HA-tagged NiR1 after treatment with 50 μM ABA for 3 h to induce endogenous NO accumulation.

### 4.6. Measurement of Endogenous NO Content

The endogenous levels of NO in shoots were determined by staining with 10 μM DAF-FM DA fluorescein, as described in [29], with some modifications. Fluorescence was detected by fluorescence microscopy with a MacroFluo (MZZ16F Leica, Wetzlar, Germany) equipped with a digital DFC300 FX Leica camera, using unchanged parameters for every measurement. Quantification was performed by counting green pixels in at least four images for tested conditions. Relative values represent the mean ± standard error of three independent biological replicates.

### 4.7. Protein Sequence Analyses and 3D Structure Modelling

Multiple protein sequence alignments were performed by using the Clustal Omega (https://www.ebi.ac.uk/Tools/msa/clustalo/ Hinxton, Cambridge, UK). Protein Database Bank (https://www.rcsb.org/) files were processed with Yasara software (www.yasara.org/).

## 5. Conclusions

The CRISPR/Cas9-mediated generation and further phenotypical characterization of *nir1-1* mutant plants made it possible to raise several basic conclusions: (1) The direct involvement of NiR1 in the biosynthesis of NO can be ruled out. (2) Blocking nitrite reduction to ammonium in Arabidopsis plants containing a single nitrite reductase led to a strong increase in NO instead of nitrite, thus severely arresting post-germination growth and further seedling establishment. (3) This situation would be similar for crops with a single nitrite reductase, but different for very important crops such as those with genomes containing two NiR orthologues that introduce redundancy. (4) nitrite reduction to ammonium is a key step not only for proper nitrogen assimilation but also for NO production, with both factors being relevant for determining plant growth and pointing to NiRs as potential targets for biotechnological applications.

Multiple post-translational modifications have been identified in NiR1. Nitration of Y has been identified in residues located at the outermost part of the protein and far from the catalytic site. However, potential effects of Y nitration on NiR1 activity due to conformational changes of the protein can not be ruled out. Ubiquitylation of K residues does not seem to affect the protein stability but more likely the subcellular re-localization, thus affecting either the activity of the enzyme or the potential to undergo other post-translational modifications. Regarding this, the identification of S-nitrosylated C amino acids involved in binding the 4Fe-4S cluster very likely avoids the binding of the cofactor, thus leading to the inactivation of the enzyme. This process may represent a regulatory loop mechanism by which NO would induce growth arrest during transient responses to stress. These identified nitration, ubiquitylation and S-nitrosylation sites in NiR1 not only help to explain some physiological responses of the plants but also allow the identification of potential targets for biotechnological approaches based on the mutagenesis of key amino acids, a strategy that can be now easily implemented by CRISPR-mediated genetic edition in crop plants.

## Supplementary Materials

Supplementary materials can be found at https://www.mdpi.com/1422-0067/21/19/7270/s1, Figure S1: Phylogenetric tree of plant orthologues of Arabidopsis NiR1; Figure S2: Generation of *nir1-1* mutant plants by CRISPR/Cas9 technology; Table S1: List of NiR1 (Q39161) peptides identified in *35S:3xHA-NiR1* plants carrying nitration, S-nitrosylation or ubiquitylation sites; Table S2: In silico prediction of ubiquitylation sites in Arabidopsis NiR1 protein; Table S3: Orthologues of Arabidopsis NiR1 in phosynthetic organisms; Table S4: Oligonucleotides used in this work.
