# Supplementary material for: Nitrite Reductase 1 Is a Target of Nitric Oxide-Mediated Post-Translational Modifications and Controls Nitrogen Flux and Growth in Arabidopsis
Source: Int J Mol Sci. 2020 Oct 1;21(19):7270. doi: 10.3390/ijms21197270 (PMC7582248; doi:10.3390/ijms21197270)
Supplement: Supplementary file 1 [file ijms-21-07270-s001.pdf]

Supplementary data

Nitrite reductase 1 is a target of nitric oxide-mediated post-translational modifications and controls nitrogen flux and growth in Arabidopsis

Álvaro Costa-Broseta, MariCruz Castillo and José León*

Figure S1

Figure S2

Table S1

Table S2

Table S3

Table S4

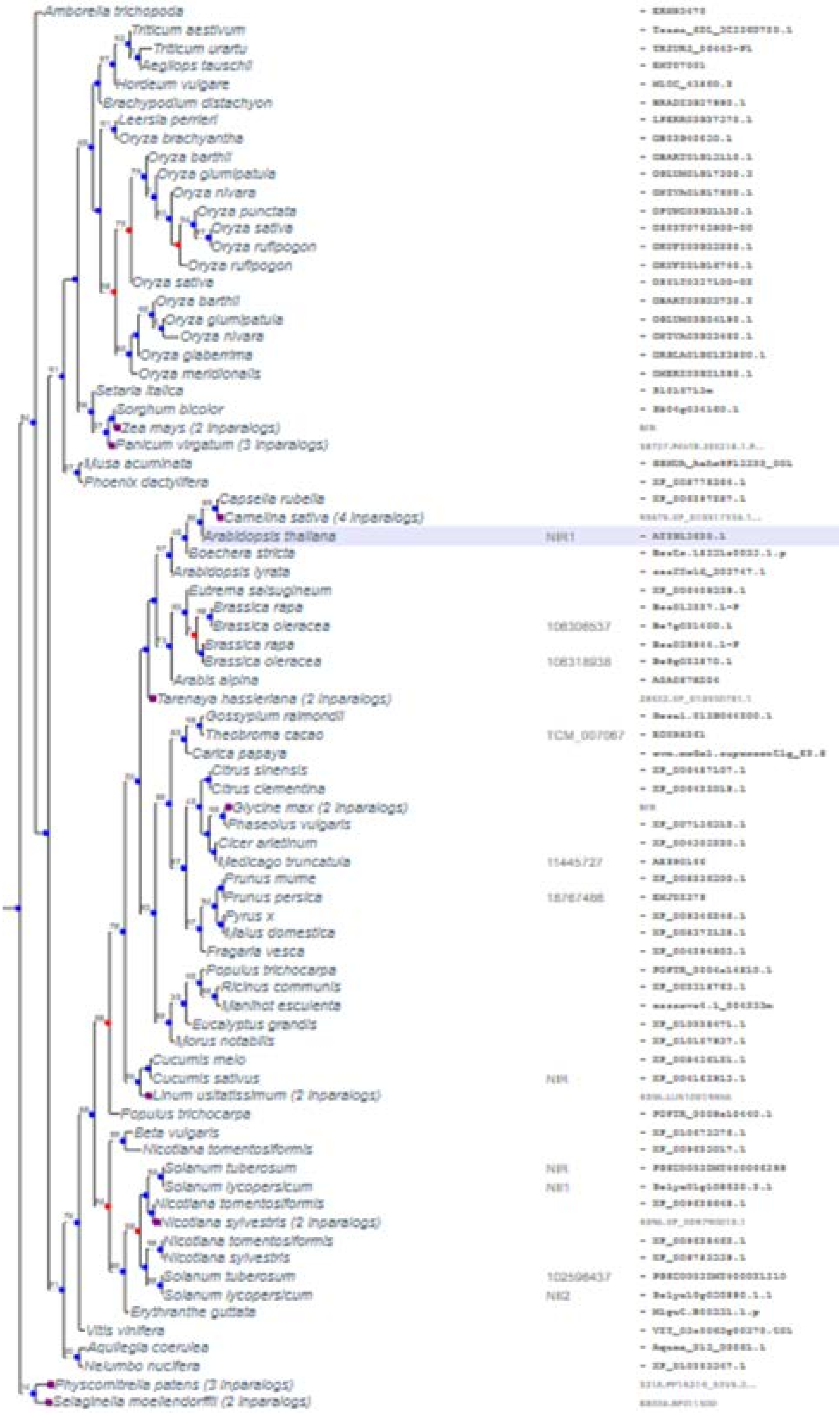

Figue S1. Phylogenetic tree of plant orthologues of Arabidopsis NiR1. Data collected from EggNOG 5.0 (http://eggnog5.embl.de/#/app/home).

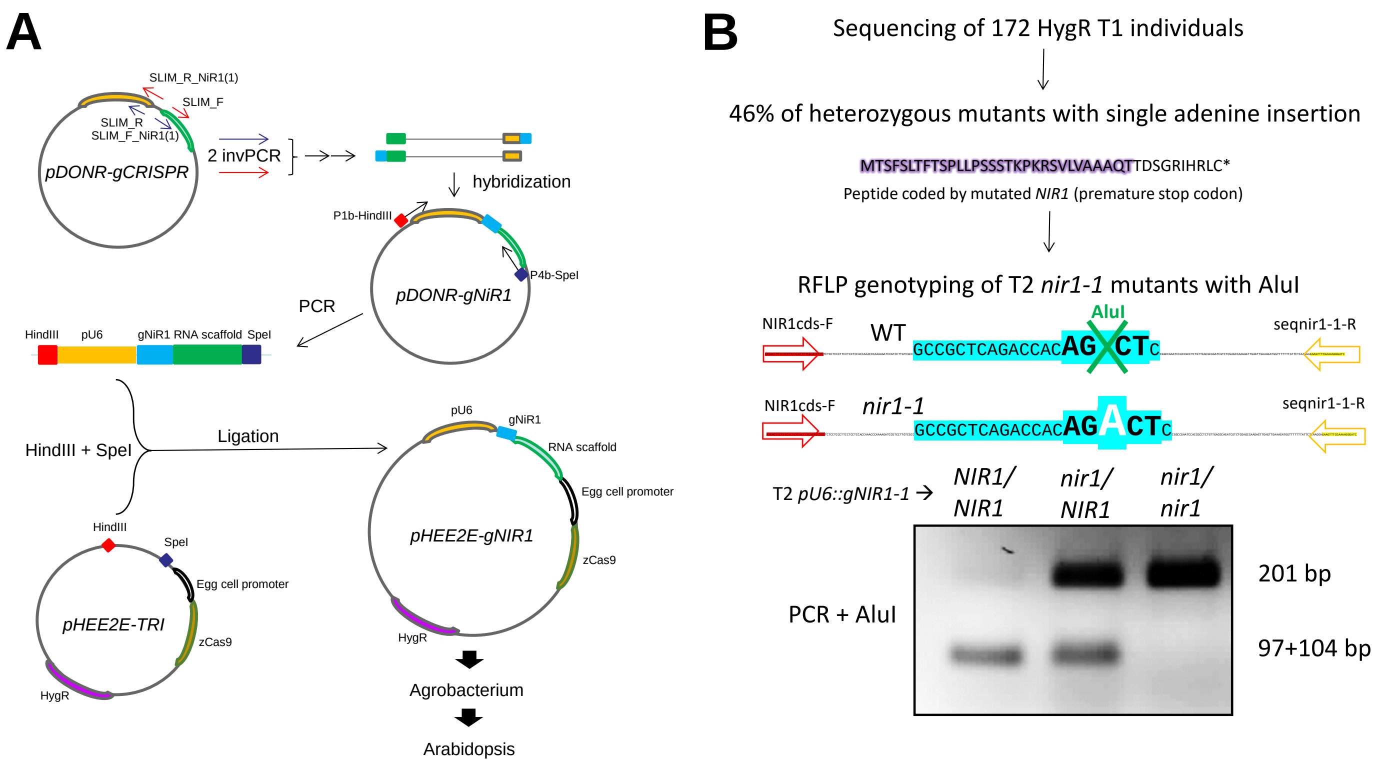

Figure S2. Generation of *nir1-1* mutant plants by CRISPR/Cas9 technology**.** A, Scheme showing the process for the generation of a transgenic plant expressing both Cas9 and a guide with a scaffold to induce a mutation in the *NIR1* locus. The NiR1 specific guide was subcloned in the pDONR207-Cas9 vector via SLIM (two separate inverse PCRs with tailed primers) and hybridization. The construction was amplified and ligated with the egg cell-specific promoter *pHEE2E-TRI* that carries the CRISPR/Cas9 technology. Such a vector was transformed into Agrobacterium and then into Arabidopsis plants. The names of the primers used for the PCRs are included. B, Scheme showing the process for the generation of a homozygous *nir1-1* mutant plant. In purple it is highlighted the region of the peptide sequence conserved from the wild type NIR1 protein. * represents the stop codon. In light blue it is highlighted the region of the *NIR1* gene corresponding to the designed specific *NIR1* guide. Bottom panel shows a 2% agarose gel electrophoresis of the genotyping via RFLP (amplifying by PCR and cutting with *Alu*I) of a wild type, heterozygous and homozygous *nir1-1* plant, all three from a second generation of transgenic *pU6::gNIR1-1* plants. The size of the bands is shown at the right side of panel.

Table S1. List of NiR1 (Q39161) peptides identified in *35S:3xHA-NiR1* plants carrying nitration, S-nitrosylation or ubiquitylation sites. Plants were treated with 50 mM ABA for 3h to generate endogenous NO accumulation. Nine independent samples were analyzed, and one miss cleavage was allowed in peptide identification.

Sequence	Modifications	XCorr	MH+ [Da]	ΔM [ppm]	Missed Cleavages
AVLEAyR	Y6(Nitro)	1,30	866,43633	-0,42	0
AVLEAyR	Y6(Nitro)	1,23	866,43590	-0,91	0
AVLEAyR	Y6(Nitro)	1,01	866,43651	-0,21	0
AVLEAyR	Y6(Nitro)	1,46	866,43620	-0,56	0
AVLEAyR	Y6(Nitro)	1,15	866,43675	0,07	0
AVLEAyR	Y6(Nitro)	1,48	866,43627	-0,49	0
AVLEAyR	Y6(Nitro)	1,48	866,43596	-0,84	0
aVLEAyRDLGTR	N-Term(Acetyl); Y6(Nitro)	1,80	1450,72693	-1,11	1
GLVAcTGSQFcGQAIIETK	C5(Nitrosyl); C11(Nitrosyl)	2,12	1983,93620	4,96	0
KyGEDGcADVTTR	Y2(Nitro); C7(Nitrosyl)	1,06	1488,59465	-4,95	1
mHWTGCPNTCGQVQVADIGFMGcLTR	N-Term(Acetyl); C23(Nitrosyl)	2,43	2896,26487	6,60	0
dGFFILk	N-Term(Acetyl); K7(GlyGly)	1,34	995,51305	-6,69	0
dGFFILk	N-Term(Acetyl); K7(GlyGly)	0,93	995,51970	0,00	0
dGFFILk	N-Term(Acetyl); K7(GlyGly)	1,26	995,51982	0,12	0
dGFFILKEk	N-Term(Acetyl); K9(GlyGly)	1,35	1252,65886	1,27	1
dGFFILkEK	N-Term(Acetyl); K7(GlyGly)	1,30	1252,65166	-4,48	1
dGFFILKEk	N-Term(Acetyl); K9(GlyGly)	1,27	1252,65203	-4,19	1
dGFFILKEk	N-Term(Acetyl); K9(GlyGly)	1,78	1252,65287	-3,52	1
FGFNLLVGGFFSPk	K14(GlyGly)	1,12	1643,86492	4,14	0
GEEGkPVEGADVYVGGR	K5(GlyGly)	2,93	1832,86455	-7,03	0
iGSDSHIGEIyKK	N-Term(Acetyl); Y11(Nitro)	1,75	1533,74663	-5,08	1
IGSDSHIGEIyKK	Y11(Nitro)	1,32	1491,75163	5,22	1
IGSDSHIGEIyKK	Y11(Nitro)	0,97	1491,75408	6,86	1
kSMEELDSEK	K1(GlyGly)	1,26	1309,59270	-1,06	1
kSMEELDSEK	K1(GlyGly)	1,23	1309,59148	-1,99	1
LADTyGSGELR	Y5(Nitro)	1,26	1226,56426	-0,46	0
LERGSSEDLVNk	K12(GlyGly)	1,14	1460,73821	2,86	1
LERGSSEDLVNk	K12(GlyGly)	1,81	1460,74248	5,79	1
lFMENGIEELAk	N-Term(Acetyl); K12(GlyGly)	1,70	1549,74907	-4,96	0
lFMENGIEELAk	N-Term(Acetyl); K12(GlyGly)	0,99	1549,75017	-4,25	0
nRFSPEPSILMk	N-Term(Acetyl); K12(GlyGly)	1,19	1574,80766	5,11	1
iGSDSHIGEIYkk	N-Term(Acetyl); K12(GlyGly); K13(GlyGly)	1,10	1716,85185	-1,94	1
RDYFGVNPQk	K10(GlyGly)	1,23	1337,66875	6,75	1

SMEELDSEk	K9(GlyGly)	0,99	1181,50725	6,88	0
SSkDDIDVR	K3(GlyGly)	1,29	1148,55217	-1,82	1
SSkDDIDVR	K3(GlyGly)	1,09	1148,56072	5,62	1
tEALLQEPFLk	N-Term(Acetyl); K11(GlyGly)	1,35	1444,77483	4,53	0
yGEDGCADVTTR	Y1(Nitro)	1,08	1331,51030	-4,95	0
yLASVIR	Y1(Nitro)	1,00	866,47185	-1,41	0
yLASVIRK	Y1(Nitro)	1,04	994,56731	-0,73	1
yLASVIRK	Y1(Nitro)	1,29	994,56713	-0,92	1
yLASVIRK	Y1(Nitro)	1,29	994,56761	-0,43	1
YLASVIRk	K8(GlyGly)	0,95	1063,62615	0,24	1
yLASVIRK	Y1(Nitro)	1,36	994,56743	-0,61	1
YLASVIRk	K8(GlyGly)	0,98	1063,62615	0,24	1
yLASVIRK	Y1(Nitro)	1,08	994,56902	0,98	1

Table S2. In silico prediction of ubiquitylation sites in Arabidopsis NiR1 protein.

Prediction was performed by using the UbPred tool (www.ubpred.org; Radivojac et al., 2010).

>SP|Q39161|NIR_ARATH FERREDOXIN--NITRITE REDUCTASE NIR1

```
MTSFSLTFTSPLLPSSSTKPKRSVLVAAAQTTAPAESTASVDADRLEPRVELKDGFFILKEKFRKGINPQ
EKVKIEREPMKLFMENGIEELAKKSMEELDSEKSSKDDIDVRLKWLGLFHRRKHQYGKFMMRLKLPNGVT
TSAQTRYLASVIRKYGEDGCADVTTRQNWQIRGVVLPDVPEILKGLASVGLTSLQSGMDNVRNPVGNPIA
GIDPEEIVDTRPYTNLLSQFITANSQGNPDFTNLPRKWNVCVVGTHDLYEHPHINDLAYMPANKDGRFGF
NLLVGGFFSPKRCEEAIPLDAWVPADDVLPLCKAVLEAYRDLGTRGNRQKTRMMWLIDELGVEGFRTEVE
KRMPNGKLERGSSEDLVNKQWERRDYFGVNPQKQEGLSFVGLHVPVGRLQADDMDELARLADTYGSGELR
LTVEQNIIIPNVETSKTEALLQEPFLKNRFSPEPSILMKGLVACTGSQFCGQAIIETKLRALKVTEEVER
LVSVPRPIRMHWTGCPNTCGQVQVADIGFMGCLTRGEEGKPVEGADVYVGGRIGSDSHIGEIYKKGVRVT
ELVPLVAEILIKEFGAVPREREENED
```

Position	Sequence	Ubiquitylation	Score
**19**	**TSPLLPSSSTKPKRSVLVAAA**	**Y**	**0.62**
**21**	**PLLPSSSTKPKRSVLVAAAQT**	**Y**	**0.68**
53	ADRLEPRVELKDGFFILKEKF	N	0.42
60	VELKDGFFILKEKFRKGINPQ	N	0.50
62	LKDGFFILKEKFRKGINPQEK	N	0.49
**65**	**GFFILKEKFRKGINPQEKVKI**	**Y**	**0.51**
**72**	**KFRKGINPQEKVKIEREPMKL**	**Y**	**0.55**
**74**	**RKGINPQEKVKIEREPMKLFM**	**Y**	**0.69**
81	EKVKIEREPMKLFMENGIEEL	N	0.45
**93**	**FMENGIEELAKKSMEELDSEK**	**Y**	**0.54**
**94**	**MENGIEELAKKSMEELDSEKS**	**Y**	**0.67**
**103**	**KKSMEELDSEKSSKDDIDVRL**	**Y**	**0.59**
**106**	**MEELDSEKSSKDDIDVRLKWL**	**Y**	**0.59**
114	SSKDDIDVRLKWLGLFHRRKH	N	0.46
**123**	**LKWLGLFHRRKHQYGKFMMRL**	**Y**	**0.54**
**128**	**LFHRRKHQYGKFMMRLKLPNG**	**Y**	**0.62**
**134**	**HQYGKFMMRLKLPNGVTTSAQ**	**Y**	**0.52**
154	QTRYLASVIRKYGEDGCADVT	N	0.36
184	VVLPDVPEILKGLASVGLTSL	N	0.45
247	GNPDFTNLPRKWNVCVVGTHD	N	0.49
274	INDLAYMPANKDGRFGFNLLV	N	0.20
**291**	**NLLVGGFFSPKRCEEAIPLDA**	**Y**	**0.69**
**313**	**VPADDVLPLCKAVLEAYRDLG**	**Y**	**0.65**
330	RDLGTRGNRQKTRMMWLIDEL	N	0.49
**351**	**GVEGFRTEVEKRMPNGKLERG**	**Y**	**0.80**
**357**	**TEVEKRMPNGKLERGSSEDLV**	**Y**	**0.80**
**369**	**ERGSSEDLVNKQWERRDYFGV**	**Y**	**0.85**
383	RRDYFGVNPQKQEGLSFVGLH	N	0.43
436	NIIIPNVETSKTEALLQEPFL	N	0.38
**447**	**TEALLQEPFLKNRFSPEPSIL**	**Y**	**0.71**

**459**	**RFSPEPSILMKGLVACTGSQF**	**Y**	**0.53**
478	QFCGQAIIETKLRALKVTEEV	N	0.17
483	AIIETKLRALKVTEEVERLVS	N	0.25
**530**	**MGCLTRGEEGKPVEGADVYVG**	**Y**	**0.57**
554	GSDSHIGEIYKKGVRVTELVP	N	0.36
555	SDSHIGEIYKKGVRVTELVPL	N	0.36
572	LVPLVAEILIKEFGAVPRERE	N	0.40

Table S3. Orthologues of Arabidopsis NiR1 in phosynthetic organisms. Data were collected from the EggNOG 5.0 database (http://eggnog5.embl.de/#/app/home).

	Orthologous sequences		
Arabidopsis thaliana (1 seqs) 3702.AT2G15620.1 ,	***Prunus persica***	**1 seqs**	3760.EMJ02379 ,
	Oryza glumipatula	**2 seqs**	40148.OGLUM01G17300.3 , 40148.OGLUM02G34190.1 ,
	Ostreococcus tauri	**1 seqs**	70448.Q010I2 ,
	Nicotiana sylvestris	**3 seqs**	4096.XP_009776470.1 , 4096.XP_009783559.1 , 4096.XP_009790010.1 ,
	Linum usitatissimum	**2 seqs**	4006.Lus10014059 , 4006.Lus10019856 ,
	Prunus mume	**1 seqs**	102107.XP_008236500.1 ,
	Oryza glaberrima	**1 seqs**	4538.ORGLA01G0123800.1 ,
	Arabidopsis lyrata	**1 seqs**	59689.scaffold_303747.1 ,
	Triticum aestivum	**1 seqs**	4565.Traes_6DL_5C536D720.1 ,
	Oryza punctata	**1 seqs**	4537.OPUNC02G31130.1 ,
	Pyrus x	**1 seqs**	225117.XP_009346246.1 ,
	Panicum virgatum	**3 seqs**	38727.Pavir.Aa00599.1.p , 38727.Pavir.Gb01294.1.p , 38727.Pavir.J03218.1.p ,
	Aquilegia coerulea	**1 seqs**	218851.Aquca_015_00061.1 ,
	Arabis alpina	**1 seqs**	50452.A0A087HD24 ,
	Cucumis melo	**1 seqs**	3656.XP_008456121.1 ,
	Theobroma cacao	**1 seqs**	3641.EOX98261 ,
	Tarenaya hassleriana	**2 seqs**	28532.XP_010530781.1 , 28532.XP_010548020.1 ,
	Zea mays	**2 seqs**	4577.GRMZM2G079381_P01 , 4577.GRMZM2G102959_P01 ,
	Oryza sativa	**2 seqs**	4530.OS01T0357100-02 , 4530.OS02T0765900-00 ,
	Citrus clementina	**1 seqs**	85681.XP_006423019.1 ,
	Nelumbo nucifera	**1 seqs**	4432.XP_010263547.1 ,
	Ricinus communis	**1 seqs**	3988.XP_002518763.1 ,
	Carica papaya	**1 seqs**	3649.evm.model.supercontig_62.8 ,
	Nicotiana tomentosiformis	**3 seqs**	4098.XP_009625017.1 , 4098.XP_009628462.1 , 4098.XP_009628648.1 ,
	Micromonas sp.	**1 seqs**	296587.XP_002507511.1 ,
	Morus notabilis	**1 seqs**	981085.XP_010107937.1 ,
	Boechera stricta	**1 seqs**	72658.Bostr.18351s0035.1.p ,
	Ostreococcus 'lucimarinus'	**1 seqs**	242159.ABO98213 ,
	Galdieria sulphuraria	**1 seqs**	130081.XP_005704382.1 ,
	Oryza brachyantha	**1 seqs**	4533.OB02G40650.1 ,
	Glycine max	**2 seqs**	3847.GLYMA02G14910.1 , 3847.GLYMA07G33570.1 ,
	Erythranthe guttata	**1 seqs**	4155.Migut.B00351.1.p ,
	Brachypodium distachyon	**1 seqs**	15368.BRADI3G57990.1 ,
	Brassica oleracea	**2 seqs**	3712.Bo7g021400.1 , 3712.Bo9g023870.1 ,
	Beta vulgaris	**1 seqs**	161934.XP_010675576.1 ,
	Cicer arietinum	**1 seqs**	3827.XP_004505220.1 ,
	Oryza barthii	**2 seqs**	65489.OBART01G15110.1 , 65489.OBART02G33730.2 ,
	Musa acuminata	**1 seqs**	4641.GSMUA_Achr9P15320_001 ,
	Bigelowiella natans	**1 seqs**	227086.JGI_V11_50588 ,
	Solanum lycopersicum	**2 seqs**	4081.Solyc01g108630.2.1 , 4081.Solyc10g050890.1.1 ,
	Solanum tuberosum	**2 seqs**	4113.PGSC0003DMT400021310 , 4113.PGSC0003DMT400066399 ,
	Triticum urartu	**1 seqs**	4572.TRIUR3_06445-P1 ,
	Coccomyxa subellipsoidea	**1 seqs**	248742.XP_005646410.1 ,
	Medicago truncatula	**1 seqs**	3880.AES90166 ,
	Malus domestica	**1 seqs**	3750.XP_008373138.1 ,
	Chlorella variabilis	**1 seqs**	554065.XP_005844715.1 ,

Fragaria vesca	**1 seqs**	57918.XP_004294803.1 ,
Sorghum bicolor	**1 seqs**	4558.Sb04g034160.1 ,
Setaria italica	**1 seqs**	4555.Si016715m ,
Chlamydomonas reinhardtii	**1 seqs**	3055.EDP00895 ,
Phoenix dactylifera	**1 seqs**	42345.XP_008778364.1 ,
Oryza rufipogon	**2 seqs**	4529.ORUFI01G16740.1 , 4529.ORUFI02G35220.1 ,
Cucumis sativus	**1 seqs**	3659.XP_004163915.1 ,
Phaseolus vulgaris	**1 seqs**	3885.XP_007156512.1 ,
Populus trichocarpa	**1 seqs**	3694.POPTR_0004s14810.1 ,
Selaginella moellendorffii	**2 seqs**	88036.EFJ11500 , 88036.EFJ18805 ,
Micromonas pusilla	**1 seqs**	38833.XP_003057941.1 ,
Camelina sativa	**4 seqs**	90675.XP_010467309.1 , 90675.XP_010468620.1 , 90675.XP_010488973.1 , 90675.XP_010517336.1 ,
Citrus sinensis	**1 seqs**	2711.XP_006487107.1 ,
Guillardia theta	**1 seqs**	55529.EKX33329 ,
Hordeum vulgare	**1 seqs**	4513.MLOC_43860.2 ,
Manihot esculenta	**1 seqs**	3983.cassava4.1_004233m ,
Gossypium raimondii	**1 seqs**	29730.Gorai.013G044200.1 ,
Capsella rubella	**1 seqs**	81985.XP_006297287.1 ,
Oryza meridionalis	**1 seqs**	40149.OMERI02G31280.1 ,
Physcomitrella patens	**3 seqs**	3218.PP1S197_146V6.4 , 3218.PP1S214_53V6.2 , 3218.PP1S53_52V6.1 ,
Vitis vinifera	**1 seqs**	29760.VIT_03s0063g00370.t01 ,
Eucalyptus grandis	**1 seqs**	71139.XP_010028471.1 ,
Oryza nivara	**2 seqs**	4536.ONIVA01G17600.1 , 4536.ONIVA02G33460.1 ,
Aegilops tauschii	**1 seqs**	37682.EMT07001 ,
Leersia perrieri	**1 seqs**	77586.LPERR02G27570.1 ,
Eutrema salsugineum	**1 seqs**	72664.XP_006409539.1 ,
Amborella trichopoda	**1 seqs**	13333.ERM93470 ,
Brassica rapa	**2 seqs**	3711.Bra015227.1-P , 3711.Bra039844.1-P ,
Volvox carteri	**1 seqs**	3067.XP_002955216.1

Table S4. Oligonucleotides used in this work.

**Name**	**Sequence (5´to 3´)**	**AGI**	**Application**
M13-F	GTAAAACGACGGCCAG		Cloning pCR8/GW/TOPO
M13-R	CAGGAAACAGCTATGAC		Cloning pCR8/GW/TOPO
35S-seq	CCTTCGCAAGACCCTTCCTCTA		Cloning pAlligator2
NOS-term-rev	GCAAGACCGGCAACAGGATTCAATC		Cloning pAlligator2
NIR1cds-F	ATGACTTCTTTCTCTCTCACTTTCACATCTCC	AT2G15620	Cloning *NIR1*
NIR1cds-R	ATCTTCATTCTCTTCTCTTTCTCTAGGCACAG	AT2G15620	Cloning *NIR1*
seqNIR1_F	GTGGTATGGATAACGTGAG	AT2G15620	Cloning *NIR1*
SLIM_F	GTTTTAGAGCTAGAAATAGCAAG		Cloning *NIR1*guide
SLIM_R	CAATCACTACTTCGACTCT		Cloning *NIR1*guide
SLIM_F_NiR1(1)	GCCGCTCAGACCACAGCTCgttttagagctagaaatagcaag	AT2G15620	Cloning *NIR1*guide
SLIM_R_NiR1(1)	GAGCTGTGGTCTGAGCGGCcaatcactacttcgactct	AT2G15620	Cloning *NIR1*guide
pDONR_F	TCGCGTTAACGCTAGCATGGATCTC		Cloning *NIR1*guide
pDONR_R	GTAACATCAGAGATTTTGAGACAC		Cloning *NIR1*guide
P1b-HindIII	TTTTTTAAGCTTGTTTAAACAAGCTTTCGTTGAAC		Cloning *NIR1*guide
P4b-SpeI	TTTTTTACTAGTGGTTTAAACAAAAAAAGCACCG		Cloning *NIR1*guide
pU6seqF	AGGCATCGAACCTTCAAGAATTTG		Cloning *NIR1*guide
pU6seqR	CTTCTCTTCTTTCAGATTCC		Cloning *NIR1*guide
Hyg-F	CGGCGAGTACTTCTACACAGC		Genotyping *NIR1* guide
Hyg-R	CTGATCGAAAAGTTCGACAGC		Genotyping *NIR1* guide
NIR1cds-F	ATGACTTCTTTCTCTCTCACTTTCACATCTCC	AT2G15620	Genotyping *nir1-1*
seqnir1-1-R	GATCCCTTTTCGAAACTTC	AT2G15620	Genotyping *nir1-1*
